# Supplementary material for: Identification of New Substrates and Inhibitors of Human CYP2A7
Source: Molecules. 2024 May 8;29(10):2191. doi: 10.3390/molecules29102191 (PMC11123773; doi:10.3390/molecules29102191)
Supplement: Supplementary file 1 [file molecules-29-02191-s001.zip › molecules-2988450-supplementary.pdf]

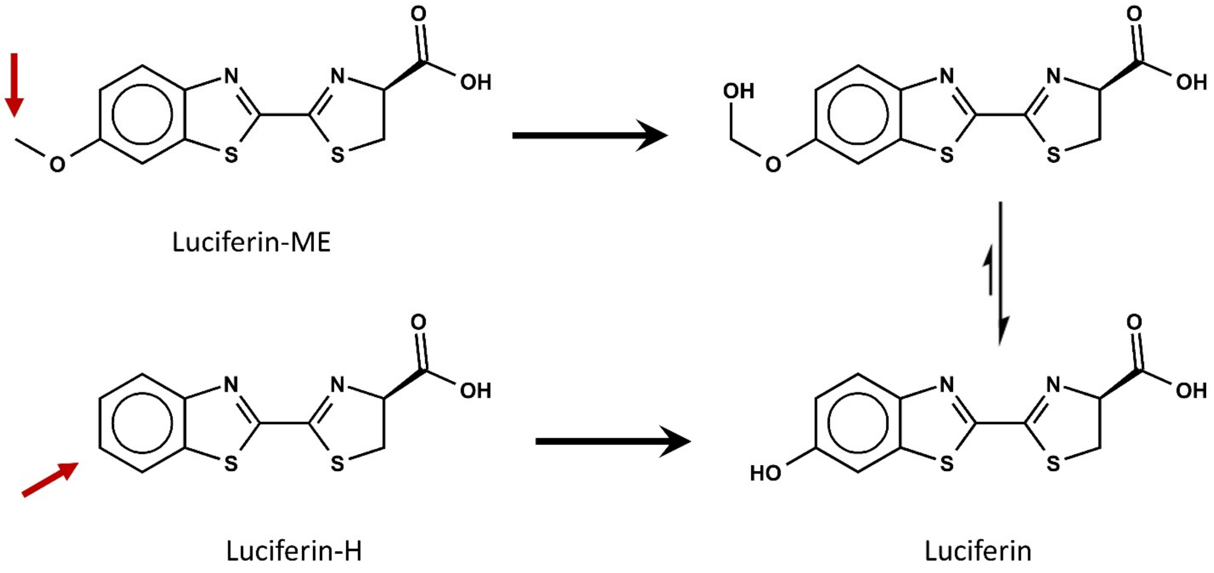

Figure S1: Scheme of the conversion of Luciferin-H and Luciferin-ME into D-luciferin. Red arrows indicate sites of oxidation by CYP enzymes that yield products for bioluminescent CYP activity detection.

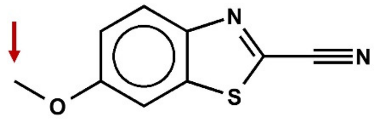

Luciferin-1A2

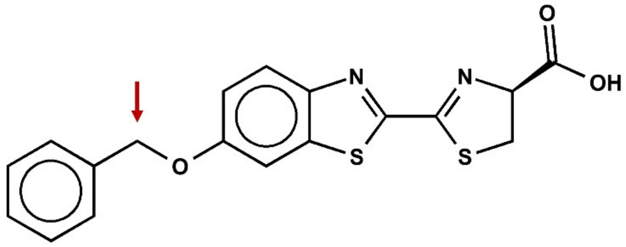

Luciferin-BE

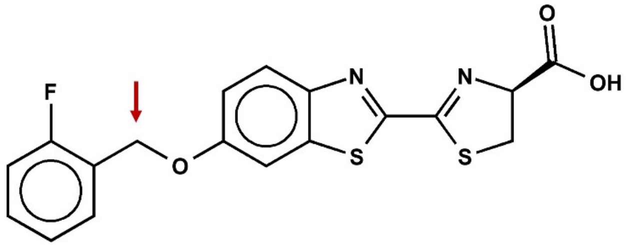

Luciferin-2FBE

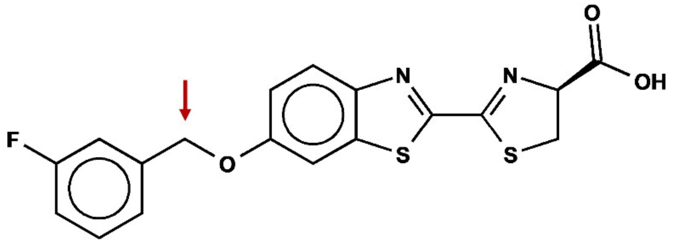

Luciferin-3FBE

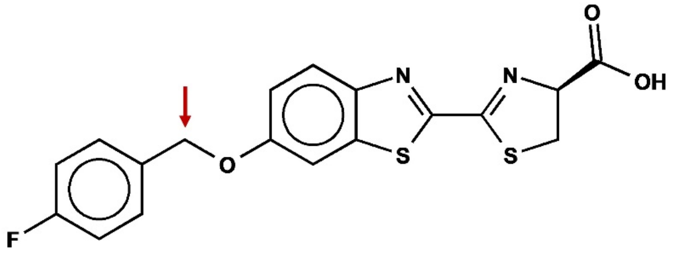

Luciferin-4FBE

Figure S2: Chemical structures of the other proluciferin substrates used in this study. Red arrows indicate sites of oxidation by CYP enzymes that yield products for bioluminescent CYP activity detection.

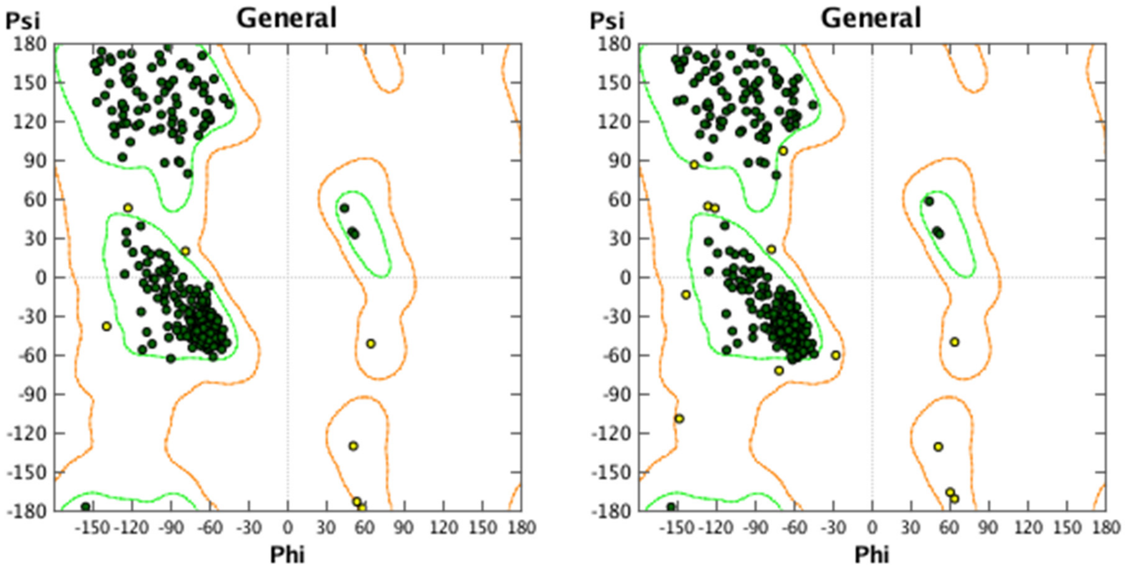

Figure S3: Ramachandran (phi-psi) plot of AlphaFold2-predicted structures of CYP2A7-WT (left) and CYP2A7 *1 (right). No outlier was shown in any of the structures.

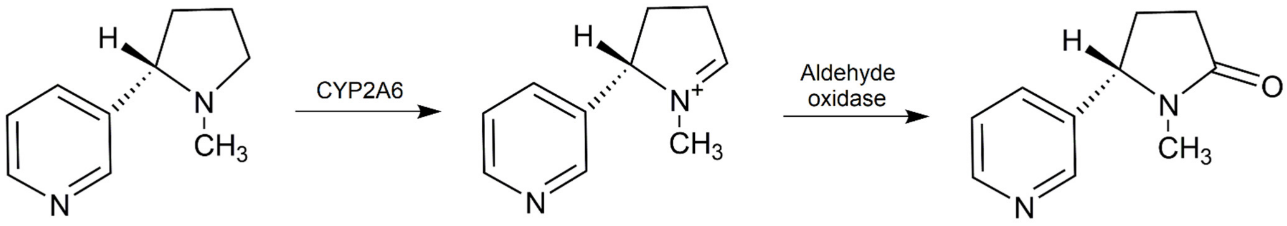

Figure S4: Scheme of the conversion of nicotine via the nicotine iminium ion to cotinine.

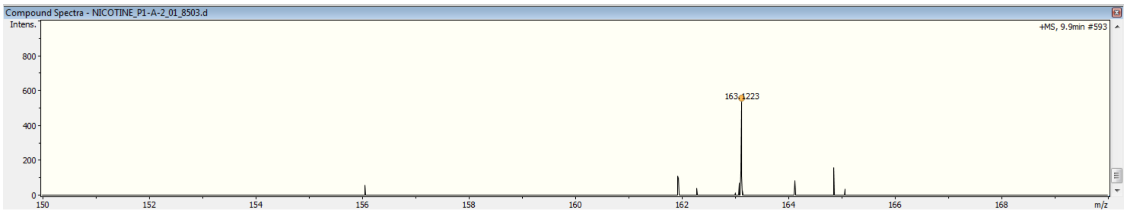

Figure S5: Mass Peak (ESI-QTOF-LCMS) of nicotine. The LC-MS system was operated in positive electrospray ionization mode at a capillary voltage of 4.5 kV.

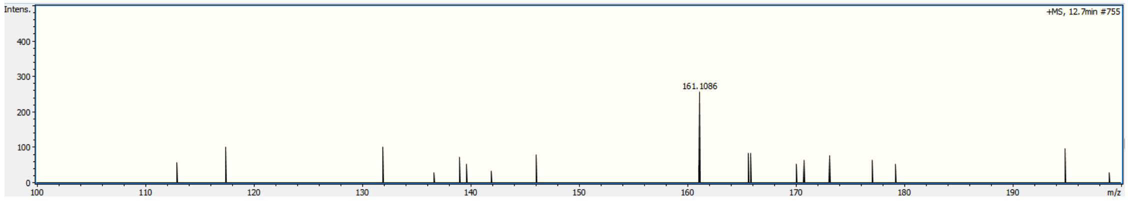

Figure S6: Mass Peak (ESI-QTOF-LCMS) of the nicotine iminium ion. The LC-MS system was operated in positive electrospray ionization mode at a capillary voltage of 4.5 kV.

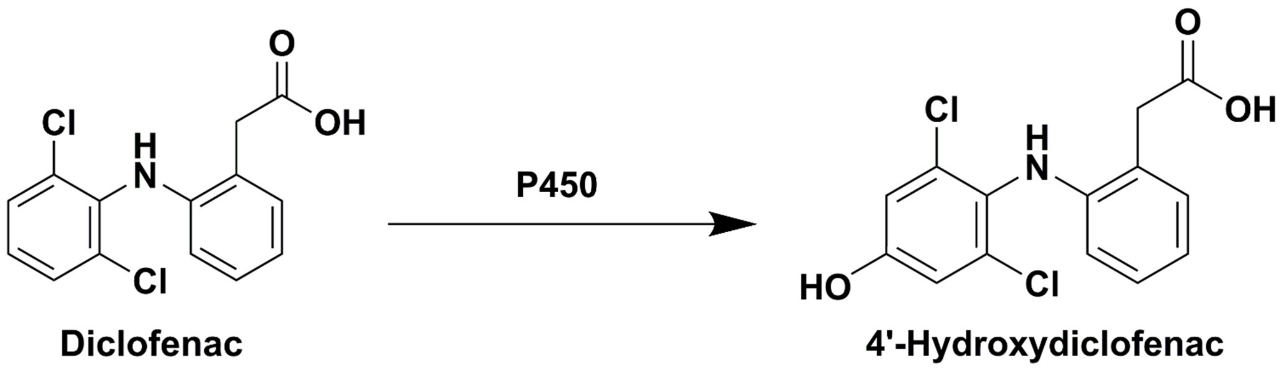

Figure S7: Scheme of the 4’-hydroxylation of diclofenac.

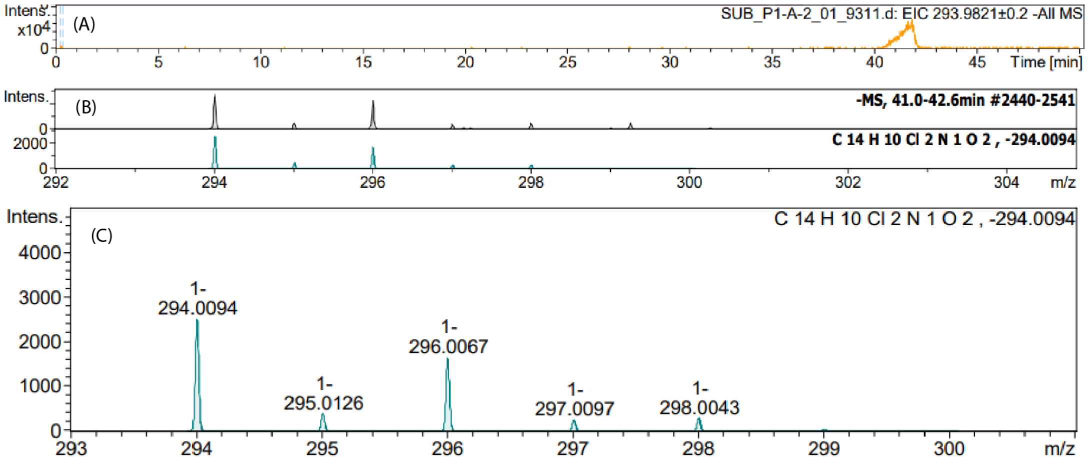

Figure S8: (A) Extracted ion chromatogram (LCMS-QTOF) of diclofenac (substrate). (B, C) Extract of the masses at a retention time 41.8 minutes. The LC-MS system was operated in negative electrospray ionization mode at a capillary voltage of 3.0 kV.

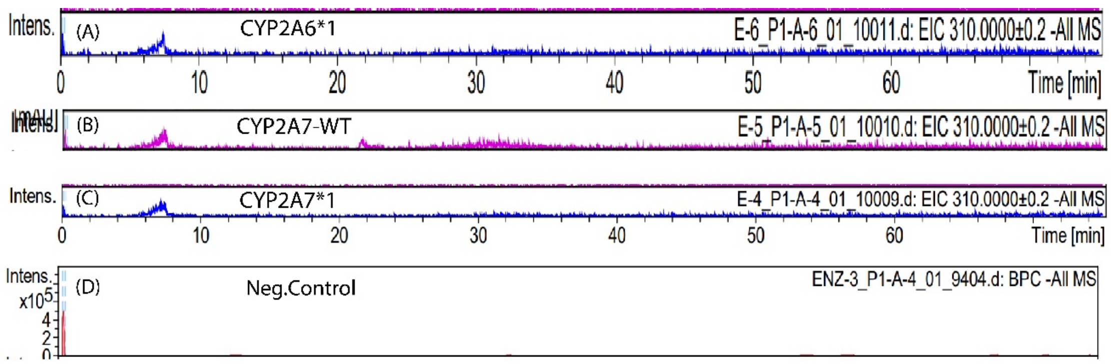

Figure S9: Extracted ion chromatograms (ESI-QTOF-LCMS) of hydroxydiclofenac by (A) CYP2A6*1, (B) CYP2A7-WT, and (C) CYP2A7-MT. (D) Negative control was strain CAD-62 (CPR only). The LC-MS system was operated in negative electrospray ionization mode at a capillary voltage of 4.5 kV.

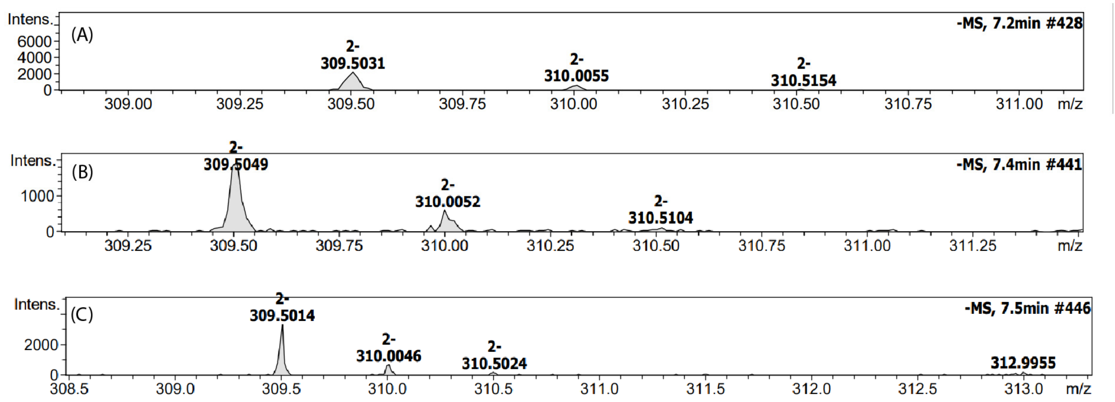

Figure S10: ESI-QTOF-LCMS chromatogram of hydroxydiclofenac produced by (A) CYP2A6*1 (B) CYP2A7-WT (C) CYP2A7*1. The LC-MS system was operated in negative electrospray ionization mode at a capillary voltage of 4.5 kV.

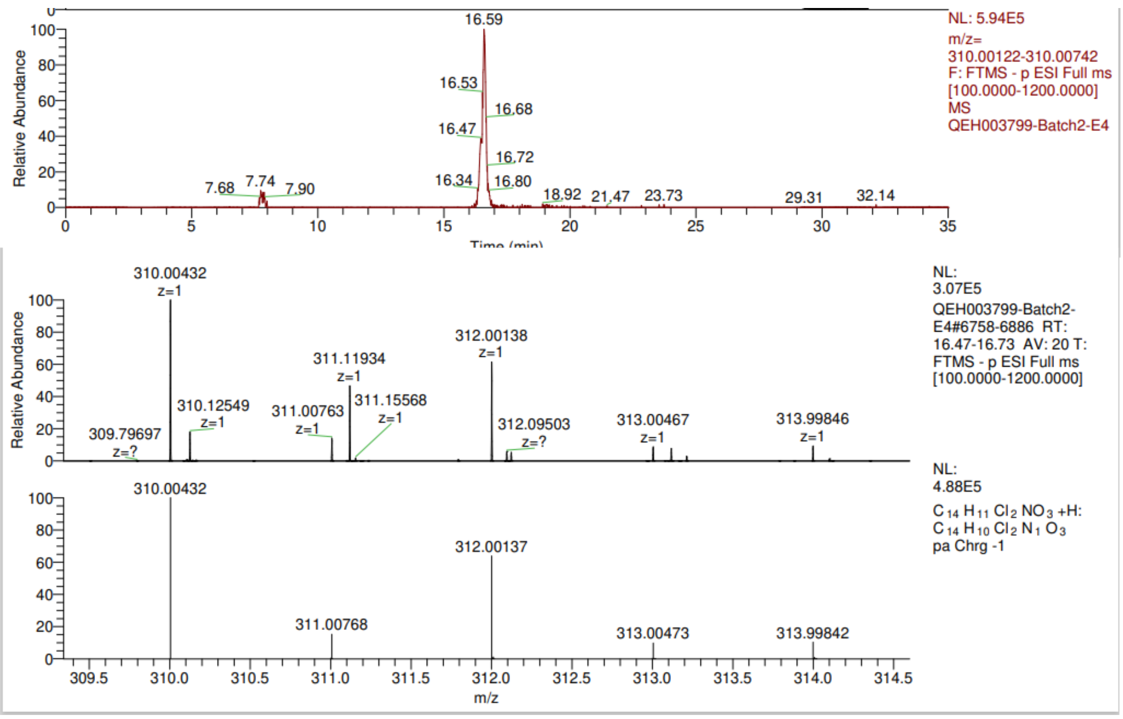

Figure S11: Extracted ion chromatogram (LC-QE-MS, $[M-H]^-$) of hydroxydiclofenac produced by enzyme bags of strain RAJ122 (expressing both CYP2A6*1 and human CPR). The reaction product ($C_{14}H_{10}Cl_2N_1O_3$) was detected at 16.59 minutes $[M-H]^-$ = 310.0043 Da (bottom).

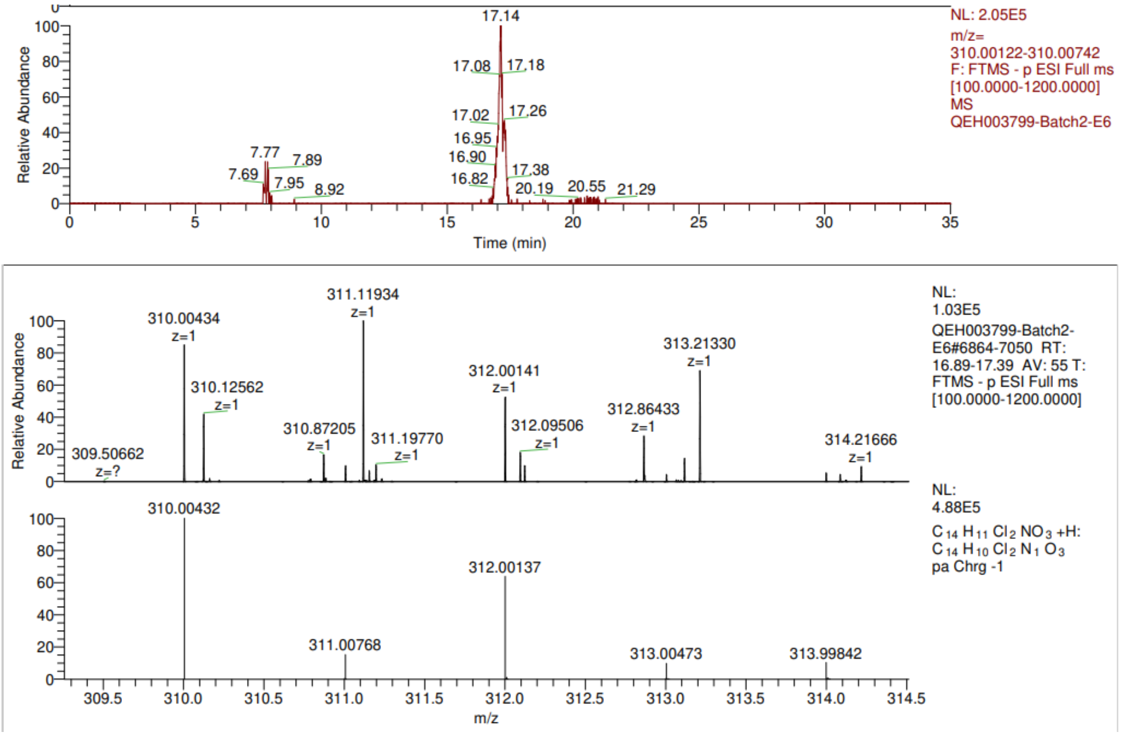

Figure S12: Extracted ion chromatogram (LC-QE-MS, $[M-H]^-$) of hydroxydiclofenac produced by enzyme bags of strain RAJ127 (expressing both CYP2A7*1 and human CPR). The reaction product ($C_{14}H_{10}Cl_2N_1O_3$) was detected at 17.14 minutes $[M-H]^-$ = 310.0043 Da (bottom).

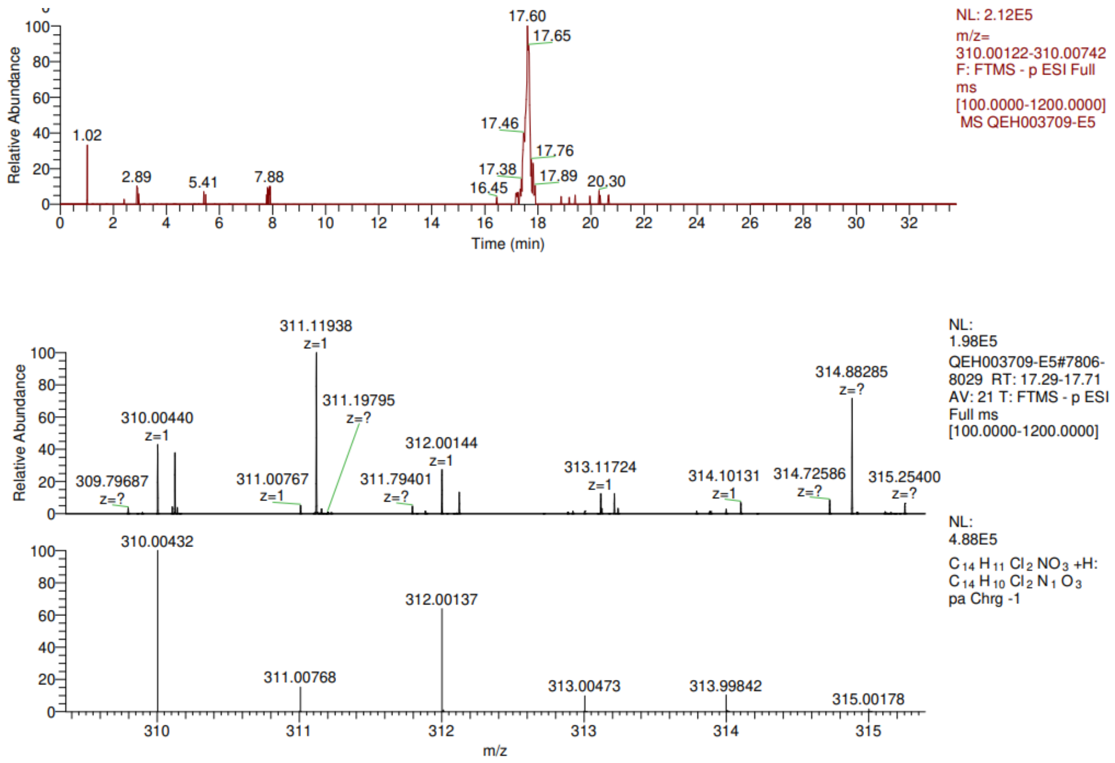

Figure S13: Extracted ion chromatogram (LC-QE-MS, $[M-H]^-$) of hydroxydiclofenac produced by enzyme bags of strain AA01 (expressing both CYP2A7-WT and human CPR). The reaction product ($C_{14}H_{10}Cl_2N_1O_3$) was detected at 17.60 minutes $[M-H]^-$ = 310.0043 Da (bottom).

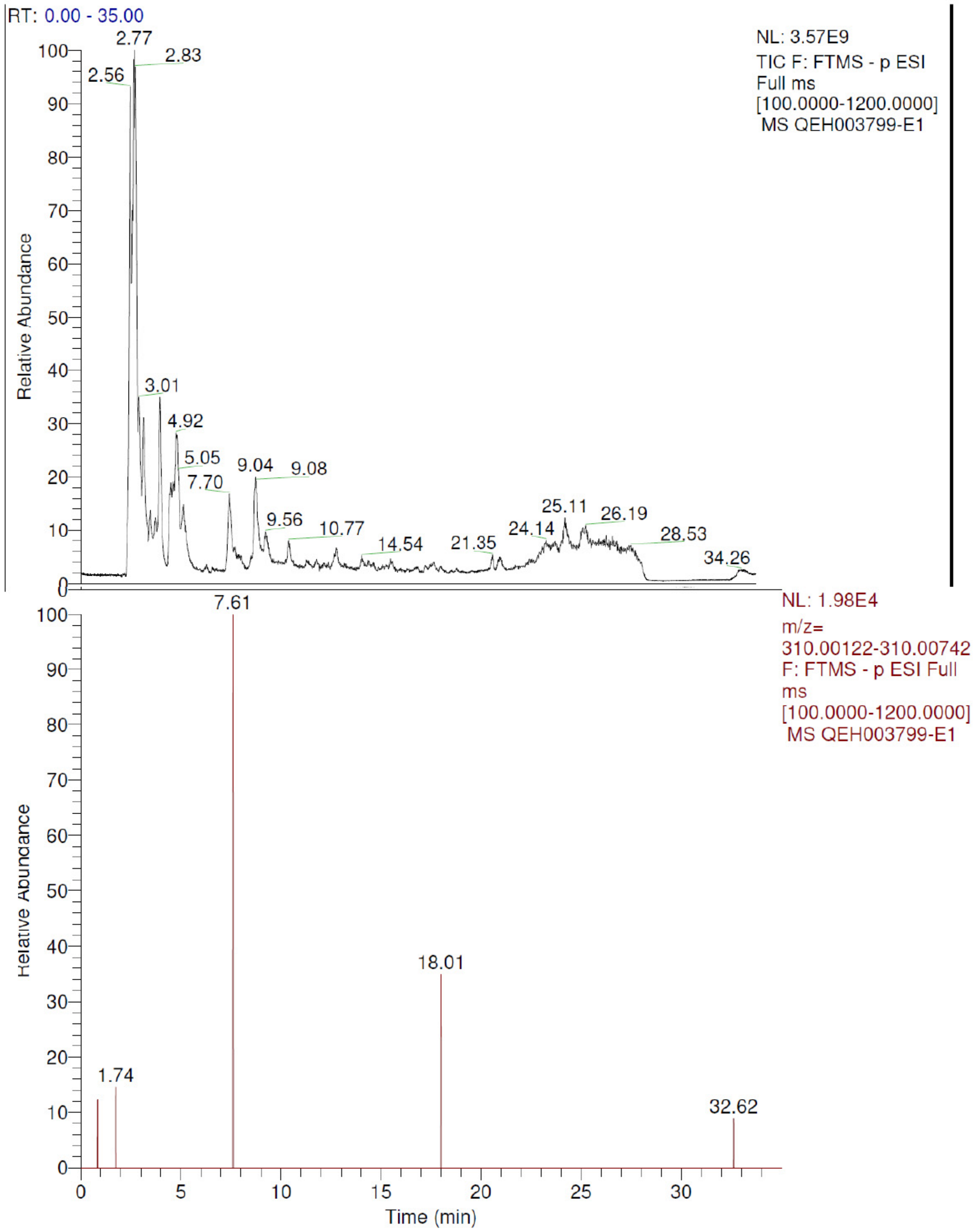

Figure S14: Extracted ion chromatogram (LC-QE-MS, $[M-H]^-$) of strain CAD62 (negative control; expressing only human CPR) showing no product formation upon incubation with diclofenac.

Table S1: Analytical data (LC/MS-QTOF) for the diclofenac metabolite found in this study

Enzyme	Metabolite	Formula	Retention time (min)	Expected mass m/z $[M-H]^-$	Observed mass m/z $[M-H]^-$	Difference Δ m/z
CYP2A6*1	Hydroxydiclofenac	$C_{14}H_{11}Cl_2NO_3$	7.2	310.0038	310.0055	5.5
CYP2A7-WT			7.4		310.0052	4.5
CYP2A7*1			7.5		310.0046	2.6

Table S2: Analytical data (LC/MS-QTOF) for diclofenac (substrate) detected in this study

Compound	Isotope peak	Formula	Retention time (min)	Expected mass m/z $[M-H]^-$	Observed mass m/z $[M-H]^-$	Difference Δ m/z
Diclofenac	Base peak	$C_{14}H_{11}Cl_2NO_2$	41.8	294.0089	294.0094	1.7
	Ist Monoisotopic peak		41.8	296.0061	296.0067	2.0
	2nd Monoisotopic peak		41.8	295.0121	295.0126	1.7

Table S3: Analytical data (LC-MS/MS-QE) for the diclofenac metabolite found in this study

Enzyme	Metabolite	Formula	Retention time (min)	Expected mass m/z $[M-H]^-$	Observed mass m/z $[M-H]^-$	Difference Δ m/z
CYP2A6*1	Hydroxydiclofenac	$C_{14}H_{11}Cl_2NO_3$	16.47	310.0038	310.0043	1.6
CYP2A7-WT			17.60		310.0043	1.6
CYP2A7*1			17.14		310.0043	1.6
